# CD4CD8αα Lymphocytes, A Novel Human Regulatory T Cell Subset Induced by Colonic Bacteria and Deficient in Patients with Inflammatory Bowel Disease

**DOI:** 10.1371/journal.pbio.1001833

**Published:** 2014-04-08

**Authors:** Guillaume Sarrabayrouse, Céline Bossard, Joe-Marc Chauvin, Anne Jarry, Guillaume Meurette, Elodie Quévrain, Chantal Bridonneau, Laurence Preisser, Karim Asehnoune, Nathalie Labarrière, Frédéric Altare, Harry Sokol, Francine Jotereau

**Affiliations:** 1 INSERM, U892, Nantes, France; 2 Université de Nantes, Nantes, France; 3 CNRS, UMR 6299, Nantes, France; 4 Department of Medicine, Division of Hematology/Oncology, University of Pittsburgh, School of Medicine, Pittsburgh, Pennsylvania, United States of America; 5 EA 4273 Biometadys, Université de Nantes, Faculté de Médecine, Nantes, France; 6 Clinique de chirurgie digestive et endocrinienne, CHU, Nantes, France; 7 INSERM UMR 913 Nantes France, Institut des Maladies de l'Appareil Digestif, CHU Nantes, Hôpital Hotel Dieu, Nantes, France; 8 ERL INSERM U1057/UMR7203, Equipe AVENIR ″Gut Microbiota and Immunity″, Faculté de Médecine Saint-Antoine, Université Pierre et Marie Curie (UPMC), Paris, Paris, France; 9 Commensal and Probiotic-Host Interactions Laboratory, UMR1319 Micalis, INRA, Jouy-en-Josas, France; 10 Université d'Angers, Angers, France; 11 EA 3826 Thérapeutiques cliniques et expérimentales des infections, Faculté de Médecine, Université de Nantes, Nantes, France; 12 Service de Gastroentérologie, Hôpital Saint-Antoine, Assistance Publique – Hôpitaux de Paris (APHP), Paris, France; National Jewish Medical and Research Center/Howard Hughes Medical Institute, United States of America

## Abstract

How the microbiota affects health and disease is a crucial question. In mice, gut *Clostridium* bacteria are potent inducers of colonic interleukin (IL)-10-producing Foxp3 regulatory T cells (Treg), which play key roles in the prevention of colitis and in systemic immunity. In humans, although gut microbiota dysbiosis is associated with immune disorders, the underlying mechanism remains unknown. In contrast with mice, the contribution of Foxp3 Treg in colitis prevention has been questioned, suggesting that other compensatory regulatory cells or mechanisms may exist. Here we addressed the regulatory role of the CD4CD8 T cells whose presence had been reported in the intestinal mucosa and blood. Using colonic lamina propria lymphocytes (LPL) and peripheral blood lymphocytes (PBL) from healthy individuals, and those with colon cancer and irritable bowel disease (IBD), we demonstrated that CD4CD8αα (DP8α) T lymphocytes expressed most of the regulatory markers and functions of Foxp3 Treg and secreted IL-10. Strikingly, DP8α LPL and PBL exhibited a highly skewed repertoire toward the recognition of *Faecalibacterium prausnitzii*, a major *Clostridium* species of the human gut microbiota, which is decreased in patients with IBD. Furthermore, the frequencies of DP8α PBL and colonic LPL were lower in patients with IBD than in healthy donors and in the healthy mucosa of patients with colon cancer, respectively. Moreover, PBL and LPL from most patients with active IBD failed to respond to *F. prausnitzii* in contrast to PBL and LPL from patients in remission and/or healthy donors. These data (i) uncover a *Clostridium*-specific IL-10-secreting Treg subset present in the human colonic LP and blood, (ii) identify *F. prausnitzii* as a major inducer of these Treg, (iii) argue that these cells contribute to the control or prevention of colitis, opening new diagnostic and therapeutic strategies for IBD, and (iv) provide new tools to address the systemic impact of both these Treg and the intestinal microbiota on the human immune homeostasis.

## Abstract 2

Gut bacterium *Faecalibacterium prausnitzii* activates a newly identified set of human IL-10-producing Treg cells (CD4CD8αα lymphocytes), revealing a mechanism by which commensal microbes contribute to host immunity.

## Author Summary

It has become evident that bacteria in our gut affect health and disease, but less is known about how they do this. Recent studies in mice showed that gut Clostridium bacteria and their metabolites can activate regulatory T cells (Treg) that in turn mediate tolerance to signals that would ordinarily cause inflammation. In this study we identify a subset of human T lymphocytes, designated CD4CD8αα T cells that are present in the surface lining of the colon and in the blood. We demonstrate Treg activity and show these cells to be activated by microbiota; we identify *F. prausnitzii*, a core Clostridium strain of the human gut microbiota, as a major inducer of these Treg cells. Interestingly, there are fewer *F. prausnitzii* in individuals suffering from inflammatory bowel disease (IBD), and accordingly the CD4CD8αα T cells are decreased in the blood and gut of patients with IBD. We argue that CD4CD8αα colonic Treg probably help control or prevent IBD. These data open the road to new diagnostic and therapeutic strategies for the management of IBD and provide new tools to address the impact of the intestinal microbiota on the human immune system.

## Introduction

The gastrointestinal tract hosts a huge number of bacteria species. In mice, these bacteria play a major role in shaping local and systemic immune responses, notably by the induction of different effector and regulatory T cell subsets, whose adequate balance is required for the maintenance of gut homeostasis [1]. It may be postulated that this balance relies on the microbiota composition, as suggested by the observation that dysbiosis are frequent in chronic immune disorders, especially Inflammatory Bowel Disease (IBD) [2]–[4]. Nonetheless, data on how the microbiota composition may impact the disease process are still lacking and in particular the existence and potential role of microbiota-induced regulatory T cells (Treg) in humans remain to be addressed [5].

CD4 T cells that express the transcription factor fork head box p3 (Foxp3) are the best-known Treg. Some differentiate in the thymus in response to self-antigens and prevent self-reactive immune responses [6],[7]. Others differentiate in the periphery under various conditions including chronic challenges by non-self antigens, such as commensal bacteria, and are strong contributors to tissue homeostasis [8]. In mice, a recent study highlighted that *Clostridium* bacteria are outstanding inducers of Foxp3 Treg in the colonic mucosa [9]. Additionally, some of these Treg expressing microbiota-specific T cell receptor (TCR) suggested that their induction involved the cognate recognition of bacterial antigens [10]. Foxp3 Treg are also present in the human gut mucosa, but their exact origin, distribution, and contribution to IBD prevention remain to be elucidated. Given that individuals with FOXP3 mutations do not always develop colitis and that intestinal inflammation is not associated with a decrease in the number of Foxp3 Treg, it has been postulated that non-Foxp3 Treg or other suppressive mechanisms may regulate colon immune homeostasis in humans [11],[12].

Interleukin (IL)-10-secreting Foxp3 negative lymphocytes correspond to a heterogeneous population that remains poorly characterized, especially in humans [13],[14]. In mice, several subsets of IL-10-secreting lymphocytes have been described in the small intestine [1], among which are double positive CD4CD8αα intraepithelial lymphocytes (IELs), which may prevent Th1-induced intestinal inflammation, in an IL-10-dependent manner [15]. In contrast, these cells are absent in the lamina propria (LP) of the mouse colonic mucosa, where most IL-10-secreting lymphocytes express Foxp3 [16]. In humans, genetic studies pointed to an important role of IL-10 in the prevention of intestinal inflammation [17],[18], but the abundance and distribution of IL-10-secreting non-Foxp3 and Foxp3 lymphocytes in the gut and the relative contribution of these cells to gut homeostasis remain poorly documented.

At least two subsets of double positive (DP) T lymphocytes, CD4CD8αβ^high^ and CD4CD8αα (CD8α^low^ lacking CD8β) have been described in the human blood [19],[20]. The latter subset has also been described in the intestinal mucosa of patients with and without IBD where it is relatively abundant compared with blood [21],[22]. However, the functional significance of these DP subsets has remained unclear to date, and, in particular, a putative regulatory role of these cells has not been studied.

Here we showed that double positive CD4CD8αα (DP8α) T lymphocytes from the human colonic lamina propria (DP8α LPL) and blood (DP8α peripheral blood lymphocytes [PBL]) represent a yet undescribed subset of Foxp3-negative T lymphocytes that share all the regulatory functions of Foxp3 Treg and secrete IL-10. We also showed that these cells are decreased in the blood of patients with IBD, as compared with healthy donors, and in the colonic mucosa of patients with IBD compared with the healthy colonic mucosa of patients with colon cancer (CC). Importantly, we identified the induction of DP8α Treg by the gut commensal bacteria *F. prausnitzii* (F) as one of the mechanisms that may explain the impact of a balanced microbiota on the prevention or the control of IBD in particular, and on human health in general.

## Results

### Double Positive CD4CD8αα T lymphocytes Are Frequently Found in the Human Colonic Lamina Propria

**Figure 1:**
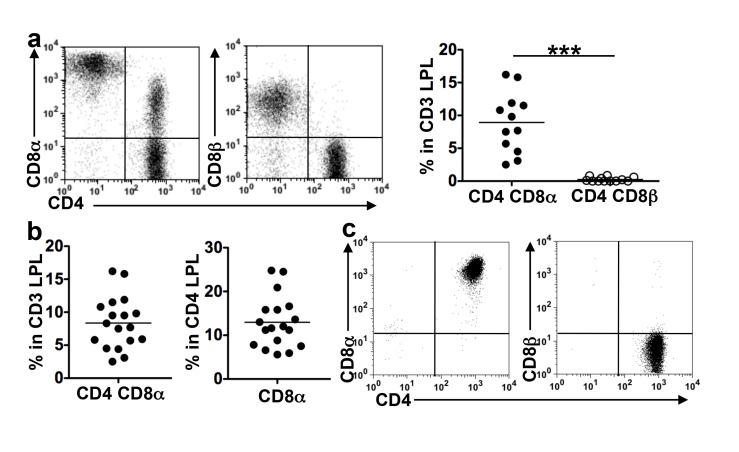
DP8α are frequently found in the *lamina propria* of healthy colon mucosa. Freshly dissociated CD3 LPL and cell lines were analyzed by flow cytometry for the co-expression of CD4 and either CD8α or CD8β. (**a**) Representative dot plots and frequencies of CD4 T cells co-expressing CD8α or CD8β among the CD3 LPL from 12 donors; ***p<0.001 (paired *t*-test). (**b**) Frequencies of CD4CD8αα cells among the CD3 or CD3CD4 LPL (*n* = 18). (**c**) Stable co-expression of CD4 and CD8α but not CD8β, by a cell line (representative of four) obtained from FACS-sorted DP8α colonic LPL, after several transfers in culture.

We analyzed the co-expression of CD4 and either CD8α or CD8β by T cells isolated from the epithelium or LP of healthy colonic mucosa from patients with CC. A significant fraction of CD3 LPL co-expressed CD4 and CD8α but not CD8β. The CD8α level expressed by these cells was variable and lower than that on CD8αβ T cells (Figure 1a). CD4CD8αα LPL, hereafter referred to as DP8α LPL, were then quantified (Figure 1b). They made up a mean of 8.5% (range 3.1–16.2) of CD3 LPL and 13.3% (range 5.9–24.8) of CD4 LPL. In the epithelium, smaller fractions of T cells co-expressed CD4 and CD8α (mean 2.4%, range 0.7–5.6), and some of these cells expressed high levels of CD8α or CD8β (Figure S2), likely corresponding to the CD4CD8αβ colonic IEL subset that we previously described [23].

To assess the stability and polyclonality of DP8α LPL, LPL populations freshly dissociated from four donors were expanded by a single polyclonal stimulation. The populations obtained contained similar fractions of DP8α cells as the original LPL (unpublished data). The CD4 (CD8α neg) and the CD4CD8α cells were then FACS-sorted, to derive pure DP8α and CD4 LPL lines by polyclonal expansion. At any time during culture the phenotype of the sorted cell lines was unchanged (Figure 1c and unpublished data). We then asked the degree of polyclonality of DP8α LPL. Two of these LPL lines (C101 and C114), obtained by a single stimulation of freshly isolated LPL expressed, respectively, 11 and 21 Vβ chains (out of the 25 tested). Moreover, DP8α LPL sorted from freshly dissociated LPL from one donor (C150) expressed 22 out of the 25 Vβ analyzed (Table S1). Therefore, DP8α T cells represent in vivo an abundant and polyclonal subset of colonic LPL, distinct from the CD4 subset by a stable expression of CD8α.

### DP8α Colonic LPL Exhibit a Treg Phenotype and Functions

**Figure 2:**
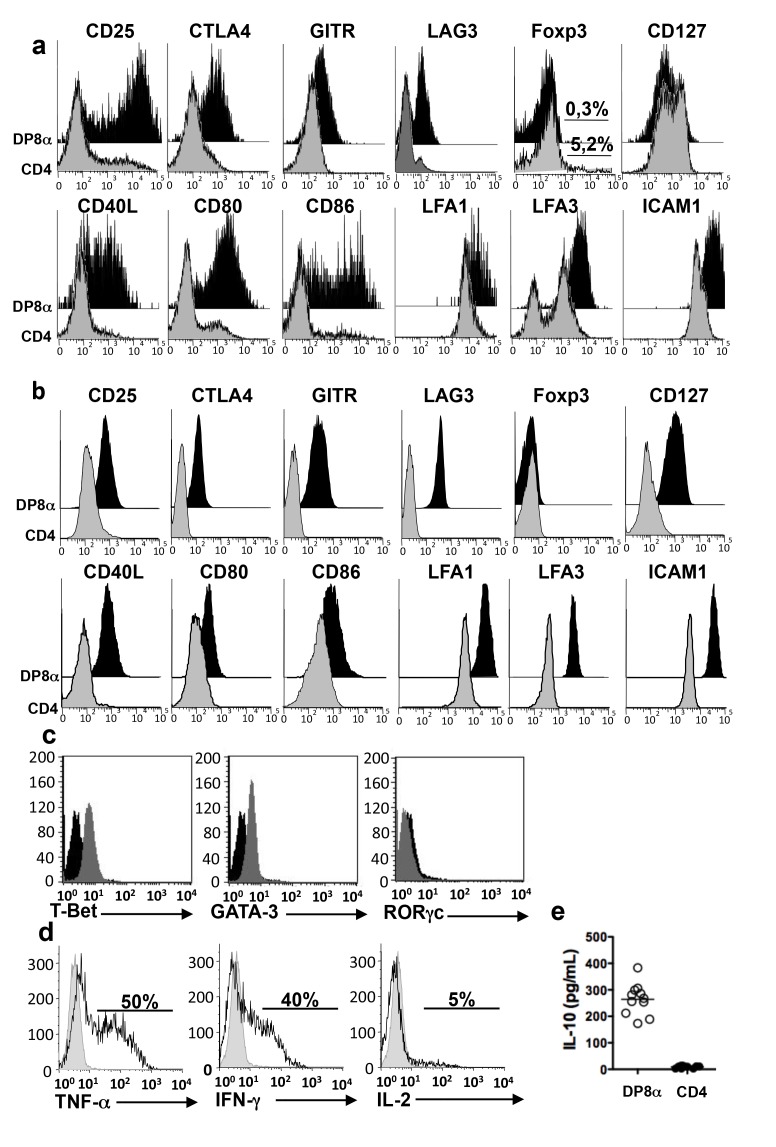
Regulatory phenotype and cytokine profile of DP8α LPL. (a) Comparison of the phenotypes of DP8α and CD4 lymphocytes among CD3 LPL freshly dissociated from healthy colonic mucosa (representative of three) (b) Phenotypes of autologous DP8α and CD4 LPL lines (representative of three pairs). (c) Expression of transcription factors by a DP8α cell line (representative of three) measured by intracellular labelling. (d) Intracellular staining for TNF-α, IFN-γ, and IL-2 in a DP8α LPL line (representative of three) stimulated for 5 h with an anti-CD3 antibody in the presence of BFA. (e) IL-10 in the supernatants of DP8α (white circles) and CD4 (black circles) LPL lines (*n* = 3) activated by an anti-CD3 antibody for 48 h as measured by ELISA (three experiments in triplicate for each cell line).

It had been hypothesized that CD4CD8 gut lymphocytes might be regulatory cells [22]. We therefore asked whether DP8α colonic LPL had a Treg phenotype, compared with their CD4 homologues. Both freshly dissociated DP8α LPL (Figure 2a) and DP8α LPL lines (Figure 2b) exhibited the same Treg phenotype. They differed from their autologous CD4 counterparts by the expression or over-expression of Foxp3-Treg markers (e.g., CD25, CTLA4, GITR, LAG-3), activation and co-stimulation markers (e.g., CD80, CD86, CD40L), and adhesion markers (e.g., LFA-1, LFA3, and ICAM-1). However, in contrast to Foxp3-Treg, they lacked Foxp3 and, as cell lines, they expressed the IL-7R (CD127). The DP8α LPL lines lacked the gut homing/localization molecules (CCR9, α4β7, and CD103). In contrast, these molecules were expressed by a significant fraction of freshly dissociated DP8α LPL (Figure S3a).

**Figure 3:**
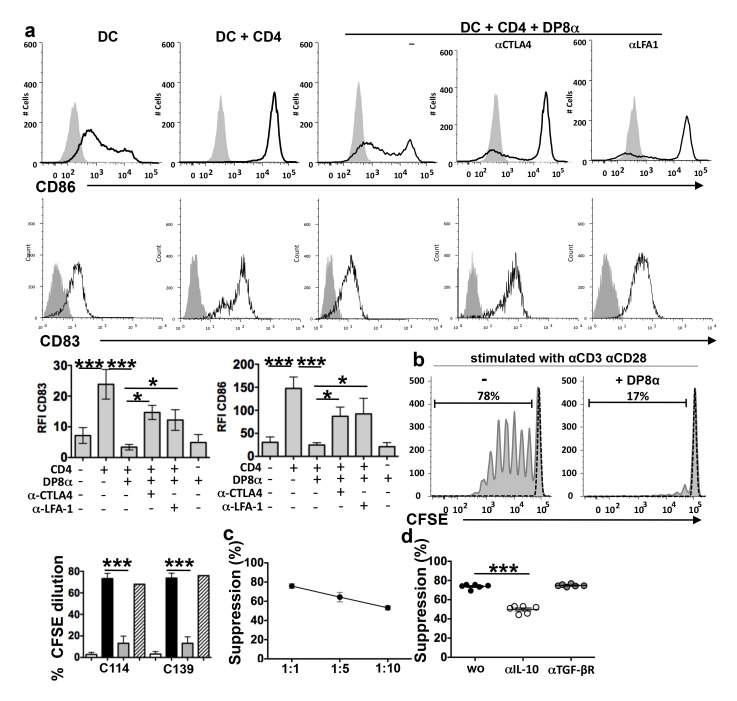
Regulatory functions of DP8α LPL lines. (a) DP8α LPL lines blocked the maturation of immature DC induced by activated CD4 lymphocytes, as shown by the inhibition of CD83 and CD86 up-regulation, and this inhibition was partially neutralised by anti-CTLA-4 and ant-LFA-1 antibodies. Immature DC were incubated for 5 d with CD4 PBL lines (expressing CD40L) in the presence or absence of DP8α LPL lines and anti-CTLA4 or anti-LFA-1 antibodies. The CD83 and CD86 expression levels were measured on gated CD3 negative cells: representative histograms and median for the CD83 and CD86 relative fluorescence intensity (RFI); (*n* = 6, two experiments performed with three cell lines); ***p<0.001, **p<0.01, and *p<0.05 (paired *t*-test). (b) Inhibition of the proliferative response of CD4 PBL by the DP8α LPL and CD4 LPL lines C114, C139 as measured by CFSE dilution. CD4 PBL sorted from healthy donor PBMC were stimulated with anti-CD3 and anti-CD28 in the presence or absence of DP8α LPL lines for 5 d at a ratio of 1∶1: representative cytometry data and histograms showing the CFSE dilution in CD8 negative lymphocytes: unstimulated (white histograms), stimulated (black histograms) stimulated in the presence of DP8α LPL (grey histograms), and stimulated in the presence of CD4 LPL (hachured histograms) (*n* = 12: six experiments done with two DP8α LPL lines); ***p<0.001 (*paired t*-test). (Only one experiment performed with the CD4 LPL lines) (c) Percent inhibition of CD4 lymphocyte proliferation by DP8α LPL lines at the indicated E∶T ratios. (d) Percent suppression of CD4 lymphocyte proliferation by DP8α LPL at a ratio of 1∶1 in the presence or absence of anti-IL-10 or anti-TGF-βR blocking antibodies; ***p<0.001 (paired *t*-test).

To further characterize these cells, we studied their cytokine profile. The DP8α LPL lines and freshly dissociated DP8α LPL expressed Tbet and Gata3 (Figures 2c and 3b) and lacked RORγc (Figure 2c and unpublished data) by 100% of their cells. Moreover, upon polyclonal activation, the DP8α LPL lines secreted tumour necrosis factor-alpha (TNF-α) and interferon gamma (IFN-γ) (approximately 50% cells), but little if any IL-2 (Figure 2d) and no IL-4, IL-5, IL-13, IL-17, or IL-22 (unpublished data). Ex vivo, the DP8α LPL exhibited the same cytokine profile as the DP8α cell lines (Figure S3c). Importantly, as shown by ELISA (Figure 2e) and quantitative PCR (Figure S3d), activated DP8α LPL lines, but not their CD4 counterparts, secreted IL-10. Therefore, DP8α LPL exhibit a phenotype and a cytokine profile of Treg but lack Foxp3. We then addressed the regulatory potential of these cells in vitro.

### Regulatory Properties of Human DP8α LPL

Similarly to Foxp3 Treg [24], the DP8α LPL lines inhibited the maturation of dendritic cells (DCs), as revealed by the inhibition of CD86, CD83 (Figure 3a), and CD80 (unpublished data) up-regulation, in a CTLA-4- and LFA-1-dependent manner (Figure 3a). The DP8α LPL lines also inhibited CD4 T cell proliferation induced by anti-CD3 and anti-CD28 antibody at all effector-target ratios used (Figure 3b and 3c), and this inhibition was partially blocked by an anti-IL-10 but not by an anti-transforming growth factor (TGF)-βR-antibody (Figure 3d). In contrast, the CD4 LPL lines induced DC maturation (Figure 3a) and failed to inhibit the proliferation of CD4 lymphocytes (Figures 3b and S4a). Notably, contrasting with Foxp3 and Tr1 Treg, the suppressive DP8α LPL proliferated upon CD3 activation in the absence of IL-2 addition, during the inhibition of CD4 T cell proliferation (Figure S4b).

### DP8α LPL Specifically Reacted with a Gut Commensal Bacteria: *F. prausnitzii*

**Figure 4:**
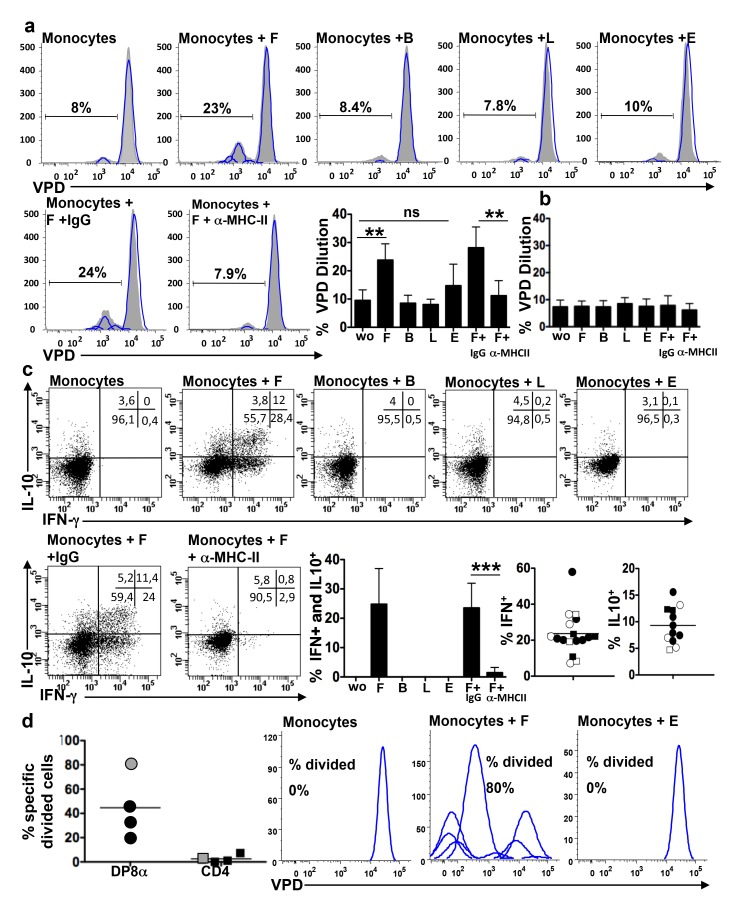
DP8α LPL specifically respond to the gut commensal bacterium *F. prausnitzii*. Flow cytometry analysis of the proliferative response (VPD dilution and FlowJo analysis) of DP8α LPL (a) or CD4 LPL lines (b) after 3 d of co-culture with allogeneic monocytes alone or loaded overnight with F in the presence or absence of an anti-MHC class-II antibody or of an irrelevant antibody (IgG), or with monocytes loaded with B, L, or E: representative cytometry data and mean percentage of VPD low cells (*n* = 6: two independent experiments performed with three DP8α LPL lines); **p<0.01 (paired *t*-test). (c) Flow cytometry analysis of the IFN-γ and IL-10 responses of DP8α LPL lines (*n* = 4) after 6 h of stimulation by monocytes, loaded overnight by F (1∶5) in the presence or absence of anti MHC class-II antibody or of an irrelevant antibody, or not loaded, or loaded with B, L, or E: representative response of the C139 DP8α LPL line and the percentages of cells secreting IL-10 or IFN- γ or both in independent experiments (two to seven experiments performed with four DP8α LPL lines: C114, black circles; C139, white circles; C192, white squares; and C140, black squares; ***p<0.001 (paired *t*-test). (d) Flow cytometry analysis of the proliferative response of freshly dissociated LPL from three donors stimulated by allogeneic monocytes (black symbols) and of sorted DP8α and CD4 LPL from one donor (grey symbols) stimulated by autologous monocytes. Responses were analyzed after 5 d of co-culture with monocytes alone or monocytes loaded overnight with F or E: mean percentage of F specific divided cells (with deduction of the divided cells to monocytes alone) (calculated on FlowJo software) and representative cytometry data of the proliferative response of the freshly sorted DP8α LPL population.

Bacteria belonging to the *Clostridium* cluster IV and XIV have been shown to induce Foxp3 Treg in the mouse colonic LP [9]. We therefore investigated if this could be the same for DP8α colonic LPL Treg. We first assessed the microbiota reactivity of DP8α LPL lines using four bacteria strains: *F. prausnitzii* (F), a major human gut bacterium of the *Clostridium* IV group, that is present at a decreased level in the faeces of patients with IBD and was shown to induce IL-10 expression by peripheral blood mononuclear cell (PBMC) [2],[25], *Bacteroides thetaiotaomicron* (B), and *Lactobacillus casei* (L), which may promote Foxp3 Treg differentiation/expansion in mice [1], and *Escherichia coli* (E), a potential pathobiont. The DP8α LPL lines and their CD4 counterparts were incubated with the bacteria alone or with a mix of allogeneic monocytes (as monocytes from the patients with cancer were usually not available) previously loaded overnight with each bacterium. The LPL lines (DP8α and CD4) did not proliferate or secrete cytokines when incubated with the bacteria alone (unpublished data). In contrast, the DP8α lines responded to monocytes loaded with F but not or much less to monocytes alone and to monocytes loaded with B, L, or E bacteria, while the CD4 LPL lines did not respond specifically to F nor to the other bacteria. This was shown both by a proliferation assay (Figure 4a and 4b) and by intracellular IFN-γ and IL-10 labelling (Figures 4c and S4c). We also observed in these experiments that DP8α LPL responses to F were suppressed by an anti-MHC class-II antibody but not by an irrelevant antibody (Figure 4a and 4c). This finding suggested that F recognition by DP8α LPL could be TCR dependent. However it could also be due to F-restricted superantigens. To further assess this important point, we compared the capacity of antibodies to CD4, CD8, MHC-I, MHC-II, HLA-DP, HLA-DQ, and HLA-DR to block the response of DP8α LPL to F presentation by a mix of three allogeneic monocytes and we analyzed the response of HLA class II-typed LPL lines to monocytes of known HLA-DP, -DQ, and -DRb1 genotypes (Table S2). As expected from TCR-dependent responses, cytokine responses of the LPL lines to F-loaded monocytes were totally inhibited by the anti-MHC-II and -CD4 but not by the anti-MHC-I and -CD8 antibodies and were inhibited at different levels by the anti-HLA-DP, -DQ, and -DR antibodies (Table 1). In addition, the LPL lines reacted to F presentation by the monocytes that shared at least one HLA class II allele but did not react with those that did not (with the exception of the response of the LPL line C192 to monocyte 5, which may be due to a cross-presentation between two similar alleles such as DQ*0301 and *0303) (Tables 2 and S2). Moreover, not supporting the superantigen hypothesis, we observed that most Vβ expressed by F-reactive DP8α LPL (identified by the co-labelling of intracellular cytokines and of Vβ in F-stimulated DP8α LPL) were also expressed by the CD4 LPL lines and that these cells did not respond to F-loaded monocytes (unpublished data). These results indicated that DP8α LPL recognized F antigens in the context of autologous HLA DP, DQ, or DR alleles and therefore in a TCR-dependent manner.

**Table 1:** HLA Class II isotype restriction of *F. prausnitzii* recognition by LPL lines.

LPL Lines	Mix of Monocytes 2, 8, and 22								
	No Bacteria	F	F+Anti MHCII	F+Anti-CD4	F+Anti-MHCI	F+Anti-CD8	F+Anti-DP	F+Anti-DQ	F+Anti-DR
C 114	1.2^a^	23.2	5.5	5.4	23.7	33.1	9.9	14	27.5
C 139	0.8	42.8	8.3	7.9	39.6	26.6	23.3	9.4	26
C 140	0.6	13.3	0.9	0.8	12.9	19.2	15.2	15.2	1.3

DP8α LPL lines (*n* = 3, C114, C139, C140) were stimulated, in the presence or not of blocking antibodies, by a mix of three allogeneic monocytes, loaded overnight by F.

a Percent IL-10 or IFN-g positive cells.

**Table 2:** Restriction of LPL responses to F by autologous HLA class II alleles.

Monocytes	LPL		
	C114	C139	C192
1	+^a^	+	+
	DP*0201^b^, DQ*0202, DR*0701	DP*0201, DP*0401	DP*0401
2	−c	+	+
	None	DP*0401	DP*0401
3	−	+	+
	None	DP*0401	DP*0401, DR*1501
4	−	−	−
	None	None	None
5	+	+	+
	DP*0201, DR*0701	DP*0201	None
6	−	−	−
	None	None	None
7	−	−	−
	None	None	None

DP8α LPL lines (*n* = 3, C114, C139, C192) were stimulated, for 6 h by allogeneic monocytes from seven HLA typed donors (Table S2) loaded overnight by F.

a Monocyte inducing a response to F.

b Shared HLA allele.

c Monocyte that did not induce a response to F.

Recent reports have indicated that IL-10-secreting Foxp3 Treg could not be induced in mice by a single Clostridium species but only by a pool of Clostridium [9],[26]. Although these inductions had not been shown so far to be antigen dependent, this finding raised the question whether F recognition by DP8α Treg was species specific or cluster specific. We assessed this using nine additional Clostridium strains, *Suboligranulum variabile*, *Roseburia intestinalis*, *C. coccoides*, *C. leptum*, *Ruminococcus gnavus*, *C. ramosum*, *C. symbosium*, *C, boltea*, and *Anaerotruncus colihominis*, which are abundant in human faeces. While the LPL lines (*n* = 3) systematically responded to F, no response to these bacteria was observed (Table 3). These results indicated that F-reactive DP8α LPL do not respond to a widely shared Clostridium antigen and thus may be F-specific.

**Table 3:** Specificity of DP8α-LPL lines and-PBL lines for *F. prausnitzii*.

Clostridium	C114	C139	C140	DTC4
*F. prausnitzii*	29^a^	22.7	46.5	26
*S. variabile*	1.3	1	0.5	0
*R. intestinalis*	1	1.2	1	0.5
*C. coccoides*	0.7	0.9	1.5	0
*C. leptum*	1	1	0.5	0.5
*R. gnavus*	0.5	0	1	1
*C. ramosum*	1.2	1	0	0.5
*C. symbosium*	1	0.5	0.5	1.2
*C. boltea*	0.5	1	1	1
*A. colihominis*	1	1	0	0.5

DP8α LPL lines (*n* = 3, C114, C139, C140) and one PBL line (DTC4) were stimulated for 6 h by monocytes, loaded overnight by different Clostridium species (1∶5).

a Percent IL-10 or IFN-γ positive cells.

We next investigated which proportion of ex vivo colonic DP8α LPL could be F-specific. To assess this, three freshly dissociated LPL populations were stimulated by a mix of F-loaded allogeneic monocytes, and pure populations of freshly sorted DP8α and CD4 LPL from one patient with colorectal cancer (C150) were stimulated by F-loaded autologous monocytes. We measured the proportions of DP8α cells, and of their CD4 counterparts, which divided in response to F-loaded but not to E-loaded monocytes nor to monocytes alone. A mean of 45% (range 20–80) of DP8α LPL divided specifically to F but not to E, while the CD4 LPL did not proliferate (Figure 4d). It has to be stressed that the fraction of F-specific cells was likely underestimated in three of these LPL populations as they were evaluated with allogeneic antigen-presenting cells (APCs) (Figure 4d black symbols). Interestingly, when autologous monocytes were used to present F to a pure population of DP8α LPL (Figure 4d, grey circle and right panels), 80% of these cells responded to F. We then assessed, with short-term cultured LPL lines C101 and C114 and with the freshly sorted DP8α LPL population (C150), the Vβ diversity of F-reactive DP8α lymphocytes. Vβ labelling showed that the F-reactive DP8α LPL expressed the majority of the Vβ of the total DP8α populations, respectively, for C101, C114, and C150 LPL: 9/11 Vβ and 1/3 Vα, 16/21 Vβ and no Vα, and 23/23 Vβ (Table S1). In sum, these data demonstrated that DP8α colonic LPL are highly polyclonal and, considering that F antigen presentation by allogeneic monocytes was suboptimal, they suggested that the TCR repertoire of these cells is highly skewed towards the recognition of *F. prausnitzii*.

### DP8α Regulatory T Cells Are Present amongst PBL

**Figure 5:**
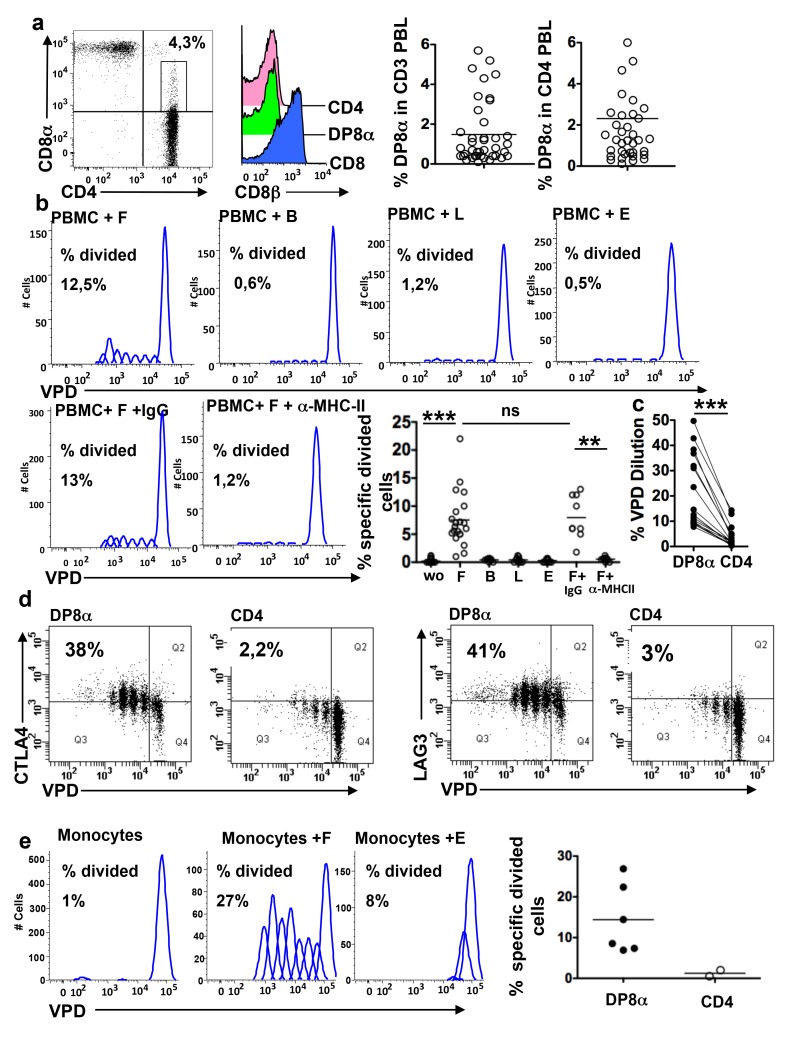
Presence of F-reactive DP8α T lymphocytes in the blood of healthy individuals. (a) Flow cytometry analysis of the frequencies of DP8α T lymphocytes in the blood of 18 donors: representative dot plot of PBMC co-labelled with anti-CD3, anti-CD4, and anti-CD8α antibodies, representative histogram of CD8β expression by gated CD8, CD4, and DP8α PBMC, and the percentages of DP8α lymphocytes among the CD3 and the CD3CD4 PBMC. (b) Proliferative responses of DP8α peripheral blood T cells to F in the presence or absence of an anti-MHC class-II antibody or an irrelevant antibody, and to B, L, and E. PBMC were cultured for 5 d with the antibody and/or indicated bacteria at a bacterium∶PBMC ratio 1∶1: representative FlowJo analysis of the VPD dilution and percentage of F-specific divided DP8α T cells in the PBMC from several donors (*n* = 18); ***p<0.001 and **p<0.01 (paired *t*-test). (c) Percent VPD dilution in paired DP8α and CD4 T cells among PBMC co-cultured with F for 5 d; ***p<0.001 (paired *t*-test). (d) CTLA4 and LAG3 expression in proliferative DP8α and CD4 PBL after 5 d of co-culture with allogeneic monocytes loaded overnight with F (*n* = 2). (e) Flow cytometry analysis of the proliferative response of freshly isolated DP8α or CD4 PBL after 5 d of co-culture with autologous monocytes alone or loaded overnight with F or E: representative cytometry data of a freshly sorted DP8α PBL population and mean percentage of F-specific divided cells (calculated on FlowJo software) (experiments performed with five freshly isolated DP8α PBL (black circles) and two CD4 PBL (white circles).

Variable proportions and distinct subsets of double positive CD4CD8 T cells have been described in the blood of patients and healthy donors among which CD4CD8αβ lymphocytes expressing high levels of CD4, CD8α and CD8β, and CD4CD8αα lymphocytes that expressed low levels of CD8α and no CD8β. Nonetheless, very little is known about the repertoire and immunological functions of these cells [19],[27]. We confirmed that CD4CD8α^low^PBL lacked CD8β and they will hereafter be referred to as DP8α PBL. DP8α PBL represented a mean of 1.5% (range 0.1%–5.7%) of CD3 PBL and of 2.3% (range 0.1%–7.8%) of CD4 PBL in healthy donors (Figures 5a and S5). It had been reported that some DP PBL represent monoclonal T cell expansions [27]. Therefore we first assessed ex vivo the polyclonality of DP8α PBL from two donors, using 25 Vβ antibodies. DP8α PBL expressed 24 out of 25 Vβ, suggesting that they were polyclonal (Table S3). To determine whether these cells could be Treg originating from the colonic mucosa, we next assessed their expression of regulatory markers (CTLA4, CD25, GITR, and LAG3), and their reactivity to F. Ex vivo, most DP8α PBLs lacked regulatory markers (unpublished data). Nonetheless, a significant percentage of these cells proliferated in an MHC class-II dependent manner among PBMC cultured for 5 d with F (mean divided cells 7.54% range 1%–22%), but not, or at much lower levels, with B, L, and E (Figure 5b). Compared with DP8α PBL, CD4 PBL yielded much lower proliferative responses to F (Figure 5c) and did not respond to E (unpublished data). Importantly, a majority of F-reactive DP8α PBL and of those that did not respond to F in the proliferation assay expressed the regulatory markers CTLA-4 and LAG-3, within 5 days of co-culture with F while their CD4 counterparts did not (Figure 5d). The fraction of DP8α PBL specific for F was re-analyzed using pure DP8α and CD4CD25^high^CD127^low^ (Foxp3^+^) PBL populations sorted from PBMC from healthy individuals stimulated by autologous monocytes loaded with F or E or not loaded. As observed previously with total PBMC, the DP8α, but not the CD4 PBL, proliferated in a specific manner to F-loaded monocytes (mean 14.4% range 7.3–26.9) (Figure 5e). Therefore the mean percent of F-specific cells among DP8α PBL may be estimated to be above 15%.

This result raised the question of whether the F-specific DP8α PBL might be a DP subset distinct from the F-non-responding cells. To assess this, we asked whether F-reactive DP8α PBL might be distinguished from the non-responding ones by their Vβ. We investigated this question using PBL lines enriched in DP8α cells (DP8) by a single sorting. In these cells lines the DP8α cells expressed 20 to 23 of the Vβ tested (Table S3). We stimulated these cell lines by monocytes loaded with F and determined the Vβ expressed by the F-reactive cells. As shown in Table S2, the F-reactive DP8α cells (VPV^low^) expressed the majority of the Vβ tested: 17/17 and 12/17. These data indicated that the repertoire of F-specific DP8α PBL is diverse and not clearly distinct from that of the total DP8α PBL population.

**Figure 6:**
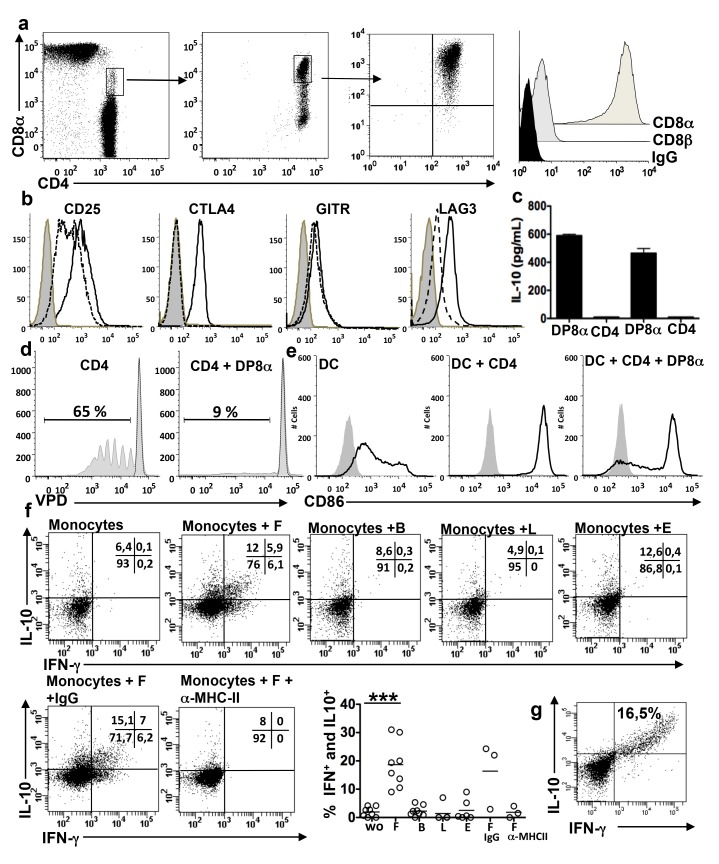
DP8α PBL lines are phenotypically and functionally similar to DP8α LPL lines. (a) Left and middle: representative staining and gating options used in the two successive sorts performed to obtain the DP8α PBL lines (*n* = 4); middle and right: phenotype of the lymphocytes obtained following polyclonal T cell expansion of the first and second sort, respectively, and representative histogram of CD8α and CD8β expression by DP8α PBL lines. (b) The DP8α PBL lines (*n* = 4) had a Treg phenotype (full line white histograms), in contrast with CD4 PBL lines (*n* = 3) (dotted line white histograms) (isotype control: grey histograms). (c) IL-10 secretion by two DP8α PBL lines and their CD4 homologues, upon stimulation with anti-CD3 antibody as measured by ELISA. (d) Representative in vitro inhibition of CD4 T lymphocyte proliferation by the DP8α PBL lines as in Figure 3b (*n* = 6: two experiments performed with three cell lines). (e) Representative inhibition of DC maturation by DP8α PBL lines as in Figure 3a (*n* = 6: two experiments performed with three cell lines). (f) IL-10 and IFN-γ responses of the DP8α PBL lines to monocytes loaded or not with F, B, L, or E, as in Figure 4c: representative dot plots and percentages of IFN-γ and IL-10 secreting cells (three experiments performed with two to three cell lines). ***p<0.001 (paired *t*-test). (g) Representative dot plot of an optimal IL-10 and IFN-γ response to F of a DP8α PBL line stimulated as in (f).

To investigate the regulatory potential of DP8α PBL, we derived pure DP8α- and, as controls, pure CD4-PBL lines, by FACS-sorting followed by polyclonal expansion, i.e., independently of any stimulation by the bacteria. We also derived two DP8α cell lines and one CD4 cell line with similar sort but starting from PBMC cultured 5 days with F. Under both conditions the first sort yielded DP8α cells with a purity of 60%–70%. Pure DP8α (Figure 6a) and CD4 PBL lines (unpublished data) were obtained after a second or a third sort and polyclonal expansions. The DP8α PBL lines lacked CD8β (Figure 6a) and expressed high levels of CD25, CTLA-4, GITR, and LAG-3 in culture (Figure 6b), and upon CD3 and CD28 stimulation secreted IL-10 (Figure 6c). In contrast, their CD4 counterparts lacked CTLA-4 and GITR, expressed CD25 and LAG-3 at lower levels (Figure 6b), and did not secrete IL-10 (Figure 6c). The DP8α PBL lines inhibited the proliferation of CD4 lymphocytes and the maturation of DCs (Figure 6d and 6e) in contrast to their CD4 homologues (unpublished data). Amongst DP8α PBL lines, between 9% and 31% of cells secreted IFN-γ and/or IL-10 in response to a mix of allogeneic monocytes (or autologous for one cell line) loaded with F but not to monocytes loaded with the other bacteria, in a MHC class-II-dependent (Figure 6f) and MHC class-II isotype-dependent (unpublished data) manner, but did not secrete IL-2 or IL-4 (unpublished data). Importantly, in some experiments all the DP8α cells that responded to F by secreting IFN-γ also secreted IL-10 (Figure 6g), suggesting that the cytokine profile of these cells is homogeneous. The CD4 PBL lines did not respond to F by proliferation or cytokine secretion (unpublished data). Therefore, DP8α PBL appear to be polyclonal and functionally homogeneous populations of circulating Treg, distinct from their CD4 counterparts as they acquired regulatory markers and functions in culture, and as a significant fraction of them reacted to F in a TCR specific manner.

### Decreased Frequency of DP8α LPL and PBL and Decreased F-Reactivity of DP8α PBL in Patients with Inflammatory Bowel Diseases Compared with Healthy Donors

**Figure 7:**
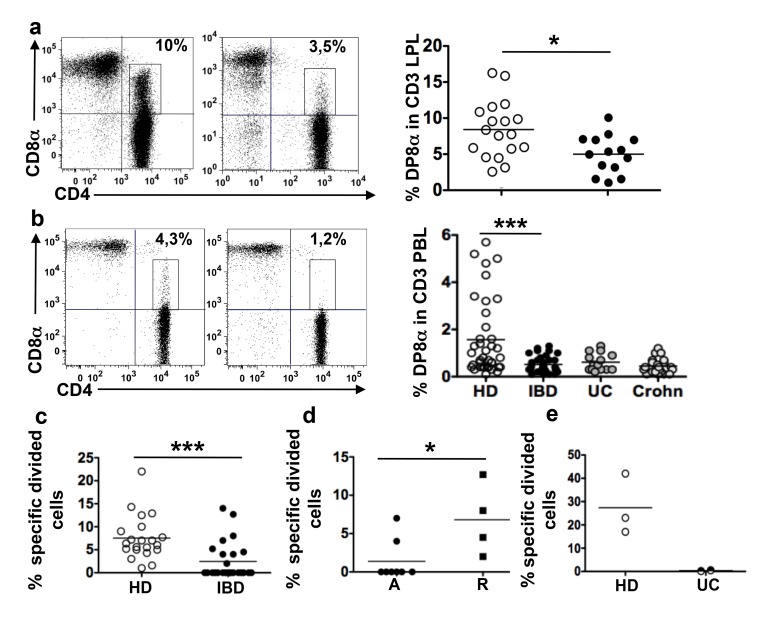
DP8α LPL and PBL, and the PBL reactivity to F, are decreased in patients with IBD. (a) Representative dot plots and frequencies of DP8α lymphocytes among CD3 LPL freshly dissociated from the inflamed mucosa of patients with IBD (right plot and black circles, *n* = 14) and healthy colon mucosa from patients with CC (left plot and white circles, *n* = 18); *p<0.05 (*t*-test). (b) Representative dot plots and frequencies of DP8α PBL in healthy donors (white circles, *n* = 38), patients with IBD (black circles, *n* = 36), patients with UC (grey circles, *n* = 14), and patients with Crohn disease (grey circles, *n* = 22); ***p<0.001 (*t*-test). (c) Flow cytometry analysis with the FlowJo software of the proliferative response (percent of F-specific divided cells) of DP8α PBMC from healthy donors (HD) (*n* = 21) or patients with IBD (*n* = 25), after 5 d of culture with F; ***p<0.001 (*t*-test). (d) As in (c) percent F-responder cells (F-specific DP8α divided cells) among PBMCs from L1 and L3 patients with Crohn disease in remission (R) and with active disease (A); *p<0.05 (Mann-Whitney test). (e) Flow cytometry analysis with the FlowJo software of the proliferative response (percent of F-specific divided cells) of DP8α lymphocytes among LPL freshly isolated from healthy mucosa (white circles, *n* = 3) and UC mucosa (black circles, *n* = 2), following stimulation for 5 d by a mix of allogeneic monocytes isolated from the blood of three healthy donors and previously incubated overnight with F.

As F is present at reduced levels in the gut microbiota of patients with IBD [2]–[4],[28],[29], we asked whether the frequencies of DP8α LPL and PBL were altered in patients with IBD compared with non-IBD donors (Tables S4 and S5 for characteristics of patients and healthy individuals). The mean frequency of DP8α lymphocytes amongst CD3 LPL was lower in the inflamed colonic mucosa of patients with IBD than in the healthy colonic mucosa of patients with CC (respectively, 4.8%, range 0.9–9.9 and 8.5%, range 3.1–16) (Figure 7a). Moreover, the mean fraction of DP8α lymphocytes amongst CD3 PBL was lower for patients with IBD than for healthy donors (respectively, 0.5%, range 0.1–1.3 and 1.5%, range 0.1–5.7) (Figure 7b). Separate analysis of the frequency of DP8α PBL in patients with Crohn disease (*n* = 20) and ulcerative colitis (UC) (*n* = 14) also indicated that in both diseases the frequency of these cells was lower than in healthy donors, respectively, mean fraction 0.4 and 0.6 range 0.1–1.2 and 0.2–1.3 (Figure 7b).

The reduced level of *F. prausnitzii* in the gut microbiota of Crohn disease [2]–[4],[28],[29] and UC patients might affect the production of F-specific Treg. To assess this hypothesis, we compared the proliferative responses to F of DP8α PBL from healthy individuals and patients with IBD. DP8α PBL from healthy donors proliferated systematically upon PBMC co-culture with F (Figure 7c), but not when cultured alone or with E (unpublished data). A similar proliferation was observed among the PBMC from nine out of 21 patients with IBD (Figure 7c). However, in the remaining 12 patients, DP8α PBL did not proliferate or proliferated only non-specifically, i.e., as much to E and/or in the absence of bacteria as to F. This lack of specific response was observed in particular with the PBMC from the five patients with UC included in this assay (unpublished data). As a result, the mean specific response to F of DP8α PBL from patients with IBD was lower than in healthy donors: respectively, mean percent divided cells among DP8α PBL, 7.5 and 2.4, ranges 1–22 and 0–14 (Figure 7c). Because patients with Crohn disease with ileal involvement (L1 and L3 stages of the Montreal classification) exhibit the clearest deficit in F in the gut microbiota, we assessed whether remission in these patients was associated with a restored frequency and/or response of DP8α PBL to F. DP8α PBL from L1 and L3 patients in remission (*n* = 4) proliferated specifically to F (mean percent divided cells: 6.4 range 2–12.7). Such a response was significantly less frequent in patients with active disease (two out of ten), mean percent F-specific divided cells 1.3 range 0–7 (Figure 7d). However, no significant difference in the frequency of DP8α circulating T cells was observed between the patients in remission and those in flair (unpublished data).

Finally, we could assess the proliferative response to F and E of LPL freshly isolated from the inflamed mucosa of two patients with UC and the IL-10/IFN-γ response of an LPL line obtained from the inflamed mucosa of another patient with UC. Similarly to what we had observed with the PBL from many patients, freshly isolated DP8α LPL (Figure 7e) and the LPL line (unpublished data) failed to respond to F in contrast to freshly sorted LPL populations (Figure 7e) and to LPL lines from healthy controls (Figure 4a).

## Discussion

Herein we demonstrated that DP8α T cells, present in the human colonic LP and blood, represent a novel subset of T cells lacking Foxp3 but sharing with Foxp3 Treg most other regulatory markers and in vitro regulatory functions, and with Tr1 Treg the capacity to secrete IL-10. We also showed that a great part of these cells specifically recognized *F. prausnitzii*, a *Clostridium* cluster IV bacterium, that is a major component of the indigenous gut microbiota frequently decreased in patients with IBD [25]. In addition, we showed that DP8α cells are decreased in the blood and colonic mucosa of patients with IBD compared with healthy individuals and the healthy colonic mucosa of patients with CC, respectively.

Together with the recent findings that bacteria of the *Clostridium* IV and XIV clusters induced the development of IL-10-secreting Foxp3 Treg in the colonic mucosa of germ-free mice and that some of these cells were specific for antigens of the inducing clostridia [9] [26], our results argue that DP8α T cells are Treg similarly induced in the human colonic mucosa through cognate activation by *F. prausnitzii* antigens. Thus, our data highlight a homology between mice and humans regarding colonic Treg induction by *Clostridium* bacteria. We also observed the presence of low fractions of DP8α lymphocytes in the epithelium of the colonic mucosa. However, as the phenotype and functions of these IEL were not characterized further, it remains unclear whether they correspond to the same Treg subset as DP8α LPL or to a colonic mucosa-specific IEL subset.

In mice, two subsets of CD4CD8αα T lymphocytes have been described in the epithelium of the small intestine [15],[30],[31]. One of these shared with DP8α LPL the capacity to secrete IL-10 and could prevent Th1-induced intestinal inflammation in an IL-10 dependent manner [15]. Interestingly studies with IL-10 reporter mice indirectly showed that this subset is absent from the mouse colonic LP, as far as all the IL-10-secreting lymphocytes of this compartment expressed Foxp3 [16]. This suggests that human DP8α and mouse Foxp3 LPL, both induced by Clostridium bacteria in the colonic mucosa, may be functional and developmental equivalents, in which case the distinct phenotype of these peripherally induced Treg populations represents a species-related divergence.

Whether Foxp3 Treg, induced by the microbiota, coexist with DP8α Treg in the human colonic mucosa is unknown. We observed that Foxp3^+^CD25^high^CD127^low^ T cells represented a much lower fraction of colonic LPL than DP8α Treg and did not react with F (unpublished data).

Another subset of CD4CD8α IEL of the mouse small intestine has been characterized recently as cytotoxic T lymphocytes. Interestingly the expression of the co-receptor CD8αα and of the cytotoxic function by CD4 IEL was shown to be associated with down-regulation of ThPOK and up-regulation of Runx 3 transcription factors [30],[31]. In contrast, the human DP8α LPL expressed ThPOK and Runx 3 at same levels as their CD4 counterparts and lacked perforin and CD107a expression (unpublished data).

Since, the induction of mouse colonic Foxp3 Treg required a mix of at least three Clostridia strains [9],[26], we asked whether nine Clostridia strains that are common in human faeces might be involved in the induction of DP8α Treg. Interestingly, four of these belonged to a mix of the 17 human Clostridial strains that enhanced Foxp3 Treg abundance in the mouse colonic mucosa [26]. None of these Clostridium strains was recognized by DP8α Treg. This showed that the target antigens of colonic DP8α Treg are not shared Clostridium antigens but rather are species specific. This further suggested that F is a major inducer of DP8α colonic Treg. However, it does not exclude that other bacteria including the Clostridium that induced Foxp3 Treg development in GF mice [26], could play a role in this induction, independently of TCR triggering. Besides cognate activation, DP8α Treg differentiation likely requires additional signals. TGF-β, vitamin A-derived retinoic acid, and tolerogenic DCs are Treg inducers constitutively present within the gut mucosa [11]. Interestingly, the production of these factors, within the mouse colon, has been shown to be stimulated by *Clostridi* [9].

Further supporting a prevalent role of F in the induction of colonic DP8α LPL, we observed that 80% of the freshly purified DP8α LPL from one donor reacted to F antigens (Figure 4d). 

Alterations in the composition of the gut microbiota and especially reduced levels of F are characteristics of patients with IBD [2]–[4],[28]. Additionally, in ileal Crohn disease, F reduction correlates with an increased risk of post-operative recurrence, suggesting that disruption of the F level is involved in the pathogenesis [2]. Importantly, the frequency of DP8α PBL and, among these, the frequency of the F-specific cells were decreased in patients with IBD compared with healthy donors. In addition, DP8α lymphocytes were less frequent in the inflamed colonic mucosa of patients with IBD compared with healthy colonic LP of patients with CC. These observations may support a role of DP8α Treg in the control of IBD. However, the decreased frequency of DP8α LPL in the colonic mucosa of patients with IBD remains to be established by comparison with healthy donors. Indeed the use of individuals with CC as a control group has two main caveats. One is the great age disparity between the patients with IBD and with CC (Table S5). The other is the potential alteration of DP8α colonic LPL frequencies, which may be associated in these patients with gut microbiota dysbiosis especially F decrease [32].

The decreased frequency of DP8α PBL in patients with IBD together with the reported decrease of F in the faeces of these patients suggests that there is a connection between reduced levels of F and decreased F-specific regulatory activity, potentially resulting in increased inflammation. However, a formal assessment needs to be done of the correlation between the levels of F and the levels of DP8a PBL and LPL in patients with IBD. Moreover, the nature of the potential connection between F and DP8α Treg levels remains unclear. F decrease might limit the differentiation or survival of DP8α Treg. Conversely, a defect in DP8α Treg differentiation or function might contribute to dysbiosis, as suggested in mice when the differentiation of Foxp3 Treg was blocked [33].

DP8α LPL and PBL, at variance with their CD4 counterparts, express high levels of the regulatory markers CD25, CTLA-4, GITR, and LAG-3, secrete IL-10, and mediate robust inhibition of CD4 T cell proliferation and of DC maturation. Therefore, these cells appear adequately fitted to inhibit effector T cell responses under both inflammatory and non-inflammatory conditions [6],[24]. These cells also expressed IFN-γ and TNF-α and Th-associated transcription factors. Similar expressions have recently been observed in mouse and human Foxp3 Treg and appear to represent phenotypic and functional specializations of these cells, paralleling those of Th lymphocytes during inflammatory responses [34]–[37]. Importantly, a number of studies have established that IFN-γ produced by Foxp3 Treg has essential immune regulatory functions [38].

Although DP8α Treg and Tr1 cells secrete IL-10, several phenotypic differences between them indicate that they represent distinct Treg populations. Among these is the expression by DP8α Treg of CD8α, high levels of CD25 and Gata-3, ex vivo and as cell lines, and the lack of expression by these cells of PD1 (unpublished data), considered to be a canonical marker of Tr1 cells by Roncarolo and colleagues [39]. In addition, both Treg seem to use different suppressive mechanisms as Tr1 suppression strongly depends on IL-10 and TGF-β activity, while these molecules, respectively, had a limited and no contribution to the suppressive activity of DP8α Treg (Figure 3d). Nonetheless, IL-10, the main inducer of Tr1 [13],[14], might also contribute to DP8α Treg induction as F is strong inducer of IL-10 secretion by peripheral mononuclear cells [2].

No clear function could be ascribed so far to CD4CD8 PBL. We showed that among these the DP8α subset lacked Treg markers ex vivo, but acquired such markers and Treg functions in culture and that a fraction of them reacted specifically to F. The fraction of F-reactive DP8α PBL seems to be lower than that of DP8α LPL. Indeed, at most 26.9% (mean 14.4%) of DP8α PBL reacted to F in an autologous antigen presentation context. As between 20% and 40% of T cells are usually non-responsive to their antigen in vitro (as observed with human clones), it may be estimated that no more than 40% of ex vivo DP8α PBL may be specific for F. Therefore, the remaining DP8α PBL should have distinct antigen specificities. Whether these cells are specific for other gut bacteria or for commensal strains present in other tissues is an interesting question. Alternatively, as DP8α PBL lacked Treg markers ex vivo, an important question was whether the fraction of these cells not-reactive to F could be effector lymphocytes expanded in response to infections, as suggested by others [20]. The observation that the majority of DP8α PBL expressed CTLA-4 and LAG-3 independently of their specificity for F, after a few days of culture with F, while very few of their CD4 counterparts expressed these markers (Figure 5d) argues that most DP8α PBL are Treg. It is likely that the circulating DP8α lymphocytes specific for F had been induced in the gut mucosa. The capacity of pTreg induced in the gut mucosa to recirculate in the blood was documented in mice by the increased frequencies of IL-10-secreting Treg outside the gut following *Clostridium* reconstitution of GF mice [9].

The frequency of DP8α PBL was significantly reduced in the blood of patients with Crohn disease and UC. In addition, in a majority of these patients DP8α PBL failed to react to F, suggesting that the decrease in circulating DP8α PBL may specifically concern the F-specific ones. Alternatively, the defective response to F might be due to immunomodulatory treatments. However, this explanation appears unlikely because this defect was not observed in some of the patients who underwent the same treatments. It will be important to investigate if the frequency of circulating F-specific DP8αTreg is correlated with the level of F and to address its prognostic and diagnostic value.

The physiological significance of circulating microbiota-reactive Treg will be important to address also in non-IBD contexts. In mice, increased resistance to experimental colitis and allergy models was observed in *Clostridium*-abundant animals. This suggests that the proportion of gut *Clostridium* may affect both colonic homeostasis and systemic immune responses and lead to conclude that *Clostridium*-induced Treg mediate these effects [9]. Likewise, the circulating F-reactive Treg identified in our study may play a role in systemic immunity. The quantification of DP8α PBL in diseases associated with microbiota imbalance should provide an answer to this question.

Importantly, the high in vitro proliferative capacity of DP8α cell lines, together with the high stability of their regulatory functions, are critical properties towards the use of these cells for adoptive transfer purposes.

In conclusion, we have identified an unanticipated new subset of Treg induced by *F. prausnitzii* that may contribute to the suppression of deleterious inflammation in humans. Importantly, these Treg exhibit a stable phenotype that allows their quantification. These results may provide new diagnostic and therapeutic strategies for IBD and systemic diseases that are associated with abnormal induction and/or function of DP8α Treg. These results should also lead to a much broader understanding of the impact of the microbiota on the human immune system than the one we currently have.

## Methods

### Cell Isolation and Cell Line Generation

PBMC were obtained from patients with IBD and healthy individuals (see Table S4 for characteristics of patients with IBD and healthy donors). Normal and inflamed colonic mucosa were obtained, respectively, from patients undergoing surgery for CC who did not undergo radiotherapy or chemotherapy, and from patients undergoing surgery for IBD (Crohn disease or UC) (see Table S5 for characteristics of patients with IBD and CC). Monocytes and PBL were obtained from healthy donor blood by elutriation (DTC platform, CHU, Nantes, France). Normal colonic mucosa was obtained from surgically resected tissue, taken approximately 10 cm downstream of the tumour. For normal mucosa, the LP was separated from the epithelium after incubation in EDTA buffer (30 min) and then minced into 1-mm^2^ fragments and washed with RPMI containing penicillin (10%) and gentamycin (0.1 mg/ml; Sigma-Aldrich). Tissue fragments were digested with collagenase and DNAse (2 mg/ml each; Sigma-Aldrich), with shaking at 37°C. Mucus and large debris were removed by filtration through a 40-µm-cell strainer (BD). Viable cells were obtained by Ficoll gradient centrifugation.

This study was approved by the ethics committee of the Comité de Protection des Personnes Ile-de-France IV (Suivithèque). All the patients signed informed consent forms. For cell line generation, CD3CD4CD8α^neg^ and CD3CD4CD8α^low^ LPL and PBL were isolated, by sorting on a FACS-Aria (Becton Dickinson). T cell lines were generated by stimulations with PHA, irradiated feeder cells, and IL-2, as described [40]. For suppression assays of T cell proliferation, CD4 T cells were isolated from PBMC using magnetic beads (130-045-101; Miltenyi). Immature DC were obtained from monocytes cultured for 5 d at 2×10^6^ cells/ml with 80 ng/ml IL-4 and 90 ng/ml GM-CSF (AbCys) in RPMI 1640 supplemented with L-glutamine, penicillin-streptomycin (10 µg/ml) (GIBCO) and 10% SVF (PAA). DC maturation was induced by co-culture with activated CD4 lymphocyte or CD4 cell lines expressing the CD40L.

### Flow Cytometry

The DP8α LPL subpopulation was identified by co-staining with PerCP-conjugated anti-CD3 (345766), FITC-conjugated anti-CD4 (555346), and APC-conjugated anti-CD8α (555369) or anti-CD8β antibodies (641058) or their isotype control. The DP8α PBL were identified by co-staining with the same anti-CD3, anti-CD4, and anti-CD8α or anti-CD8α isotype control antibodies (as above), and by gating on CD3CD4 cells expressing lower amounts of CD8α than CD8αβ T cells do. The marker combinations and gating options used for the quantification of DP8α LPL and PBL are described in Figures S1 and S5, respectively. For phenotype determination, the following antibodies were used: phycoerythrin (PE)-conjugated anti-CD25 (555432), anti-CTLA4 (555853), anti-LAG3 (514782), anti-CD40L (335853), anti-LFA1 (555384), anti-LFA3 (555921), anti-ICAM1 (555511), anti-FOXP3 (17477771), anti-GATA3 (560574), anti-TBET (125825), anti-RORγc (12698880) (all from Becton Dickinson), anti-CD80 (IM2729U), anti-CD83 (IM2218U), anti-CD86 (IM1976U), anti-GITR (FAB689), (Beckman), and PE-conjugated anti-human TCR Vβ chains (Immunotech Beckman Coulter). The following colour- and isotype-matched control antibodies were used to confirm the staining specificities: APC-conjugated mouse IgG1 (555751), PE-conjugated-mouse IgG1 (555749), and PE-conjugated-mouse-IgG2ak (555574). Single-stained beads (Comp beads; Becton Dickinson) for each fluorochrome were used for compensation settings. Cells (2×10^5^) were stained in PBS/0.1% BSA containing antibodies for 30 min at 4°C in the dark. The cells were washed and 10^4^ to 10^5^ cells were acquired in the CD3 cell gate, on a FACScalibur or a Canto II flow cytometer and analyzed using Diva or CellQuest softwares (BD). The data were further analyzed with FlowJo software (Tree Star).

### Suppressive Assays: Inhibition of CD4 Proliferation and DC Maturation

Freshly sorted CD4 PBL (5×10^4^) were incubated with 5 µM CFSE (Invitrogen) in PBS containing 0.1% BSA for 15 min, washed, and then stimulated with anti-CD3/anti-CD28 activation beads (Miltenyi) at a 1∶1 ratio, in the presence or absence of DP8α or CD4 LPL lines at the indicated effector∶target (E∶T) ratios and in the presence or absence of anti-IL-10 or anti-TGF-β antibodies. The proliferation of target CD4 T cells was assessed by flow cytometry analysis of CFSE dilution among CD8-negative T cells, on day 5. CD40L expressing CD4 LPL lines were cultured with immature DC (1∶1 ratio) for 2 to 3 d (to induce DC maturation) in the presence or absence of DP8α cell lines and of anti-CTLA-4 or anti-LFA-1 antibodies. The cells were stained with APC-conjugated anti-CD3 (555335) and PE-conjugated anti-CD80 (IM2729U) or PE-conjugated anti-CD83 (IM2218U), or PE-conjugated anti-CD86 (IM1976U) or isotype control antibodies. CD3 negative cells were analyzed by flow cytometry to determine the level of expression of CD80, CD83, and CD86.

### Intracellular Cytokine Assay

Lymphocytes were incubated 6 h with 0.1 µg/ml plate-bound CD3 antibody (OKT3 eBioscience) or, at a 1∶1 ratio, with bacteria or monocytes (when autologous monocytes were not available a mixture of monocytes from four donors were used to permit the sharing of HLA class-II alleles between the monocytes and the responding cells. Indeed, monocytes from a single donor that lacked any MHC overlap failed to induce a response to F, as shown on Table 2), previously incubated overnight with the different bacteria at a 5∶1 ratio. To prevent cytokine secretion, 10 µg/ml brefeldin A (Sigma-Aldrich) was added for the last 6 h of stimulation. Stimulated cells were stained with PerCP-conjugated anti-CD3, FITC-conjugated anti-CD4, and APC-conjugated anti-CD8α. The cells were then fixed for 10 min in PBS/4% paraformaldehyde (Sigma-Aldrich) and washed. Cytokine-specific antibodies were then added for 30 min at room temperature. Reagent dilutions and washes were performed with PBS containing 0.1% BSA and 0.1% saponin (Sigma-Aldrich). Cytokine secretion was assessed by flow cytometry in DP8α or CD4-positive LPL and cell lines. In some experiments TCR Vβ and intracellular labelling were analyzed among cytokine-labelled DP8α cell lines. The following antibodies were used: PE-conjugated anti-IL-2 (559334), anti-IL-4 (554486), anti-IL-5 (554395), anti-IL-10 (562400), anti-TNF-α (554418), and anti-IL-22 (515303) and APC-conjugated anti-IL-13 (554571), anti-IFN-γ (554551), and anti-IL-17 (51717871). We also used in some experiments anti-CD3 Brilliant Violet (562426), anti-IFN-γ APC (554702) combined with the anti-IL-10 PE. For blocking experiments, we used anti HLA class II ascites (clone 206 produced in our laboratory) and an irrelevant mouse IgG.

### IL-10 Production by ELISA

T cell lines (10^5^ in 200 µl) were stimulated with plate bound anti-CD3 antibody (OKT3, eBioscience) at 0.1 µg/ml for 2 d. The levels of IL-10 in the supernatants were measured by ELISA (R&D Systems).

### Bacterial Cultures

*F. prausnitzii* A2–165 (F) was grown for 20 h at 37°C in LYBHI medium (brain–heart infusion medium supplemented with 0.5% yeast extract; Difco), cellobiose (1 mg/ml; Sigma–Aldrich), maltose (1 mg/ml; Sigma-Aldrich), and cysteine (0.5 mg/ml; Merck) in an anaerobic chamber. *B. thetaiotaomicron* VPI-5482 (B) and *L. casei* (ATCC 393) (L) were grown for 20 h at 37°C in an anaerobic chamber in Wilkins-Chalgren medium (33 g/l; Oxoid) and LYBHI medium, respectively. *E. coli* K12 (E) was grown for 20 h at 37°C with agitation (80 rpm) in Luria-Bertani medium (20 g/l; Invitrogen). The supernatant and pellet for each bacterial strain were obtained by centrifugation at 1,700 *g* at 4°C for 15 min.

### T Lymphocyte Proliferation Assays to Bacteria

Lymphocytes (PBMC, LPL, PBL, or cell lines) were labelled for 15 min incubation at 37°C in the dark with 1 µM VPD (BD Bioscience) in PBS containing 0.1% BSA. The cells were washed twice in medium containing 10% FBS. F, B, L, and E were sonicated for 15 min at high speed and then co-cultured with VPD labelled PBMC at 1∶1 ratio, or with monocytes overnight (at a ratio 5∶1) in presence of gentamycin (0.1 mg/ml). When autologous monocytes were not available a mix of allogeneic monocytes from three to four donors was used to present the bacteria. Monocytes, loaded with bacteria or left unloaded were washed and mixed with VPD-labelled cells (1 to 1.5×10^5^) at a 1∶5 ratio. After 3 to 5 d, the proliferation of T cells was assessed by flow cytometry analysis of the VPD dilution in CD3CD4CD8a^low^ and CD3CD4CD8a^neg^ PBMC or in CD4- or in CD8α positive cells for T cell lines and the fraction of divided cells was determined by FlowJo analysis of the VPD dilution. The HLA class II dependency was analyzed by adding a specific antibody every 48 h or an irrelevant mouse IgG.

### Quantitative RT-PCR Analysis

Briefly, after cell lysis using the Trizol reagent (Life technologies), total RNA were extracted using the RNeasy Micro kit (Qiagen) and reverse transcribed using the Superscript II reverse transcriptase (Life technologies). PCR amplification was performed with an amount of cDNA corresponding to 50 ng of total RNA. For qPCR, amplification was done using iQ SYBR Green Supermix (Bio-Rad) and specific gene expression was calculated using the 2DDCT method (using GAPDH as calibrator). The primer sequences used are available on request.

### Statistical Analysis

Statistical analysis was performed with the GraphPad Prism version 5.0 (GraphPad software). Paired and unpaired *t*-tests and the Mann-Whitney test were used, as indicated in the Figures 1–7. Differences were considered significant at *p*<0.05.

## Supporting Information

Figure S1 **Marker combinations and gating options used for the quantification of DP8α cells among T lymphocytes obtained from freshly dissociated LP colonic samples.**
(TIF)

Figure S2 **DP8α among the IEL of healthy colonic mucosa.** Freshly dissociated IEL were analyzed by flow cytometry for the co-expression of CD4 and either CD8α or CD8β. Representative dot-plots and frequencies of CD4 T cells co-expressing the CD8α or CD8β among CD3 IEL from eight donors; **p<0.01 (paired *t*-test).
(TIFF)

Figure S3 **Flow cytometry analysis of the gut homing/localization markers and cytokine profile of DP8α LPL lymphocytes from healthy colonic mucosa.** (a–c) Freshly dissociated CD3 LPL. (a) Expression of gut homing markers by DP8α LPL. (b) Expression of transcription factors, as in Figure 1e. (c) Cytokines secreted upon stimulation with anti-CD3 (as in Figure 1f). (d) IL-10 m RNA expression in DP8α and CD4 LPL lines (*n* = 3).
(TIF)

Figure S4 **Lack of regulatory functions and F reactivity of CD4 LPL lines.** (a) Flow cytometry figure showing the proliferation of CD4 lymphocytes is inhibited by DP8α LPL line C139 but not by its CD4 counterparts as measured by VPD dilution. (b) Dot plot showing the proliferative response of a DP8α LPL line (representative of four) upon stimulation with an anti-CD3 antibody (measured by PKH26 dilution) in a co-culture with VPD labelled CD4 T lymphocytes. (c) Flow cytometry analysis of the intracellular cytokine response of DP8α LPL lines (*n* = 3) and autologous CD4 LPL lines (*n* = 3), as in Figure 3b; ***p<0.001 (*paired t*-test).
(TIF)

Figure S5 **Marker combinations and gating options used for the quantification of DP8α cells among freshly-isolated PBMC.**
(TIF)

Table S1 **Vβ diversity of total DP8α and F-reactive DP8α-cells among LPL lines and ex vivo LPL.** Cytometry analysis of Vβ expressed by DP8α cells among LPL lines (*n* = 2 C101, C114) stimulated# or not* by F-loaded monocytes and among freshly purified LPL (C150) stimulated## or not** by F-loaded monocytes. A gate was done on DP8α cells.
(XLSX)

Table S2 **MHC class II alleles expressed by LPL lines and monocytes.**
(XLSX)

Table S3 **Vβ diversity of total DP8α and F-reactive DP8α-cells among PBMC and PBL lines.** Cytometry analysis of Vβ expressed by DP8α cells among PBMC* (*n* = 2 DTC4, DTC28) and among VPD-labelled cell lines derived from these PBMC (by a single sorting and polyclonal expansion of DP8α cells) stimulated*** or not** by F-loaded monocytes. A gate was done on DP8α cells.
(XLSX)

Table S4 **Characteristics of the donors used to study DP8α PBL.**
(XLSX)

Table S5 **Characteristics of the donors used to study DP8α LPL.**
(XLSX)

## Abbreviations

APC: antigen presenting cell
B: *Bacteroides thetaiotaomicron*
CC: colon cancer
DC: dendritic cell
DP: double positive
DP8α: double positive CD4CD8αα
E: *Escherichia coli*
F: *Faecalibacterium prausnitzii*
IBD: Inflammatory Bowel Disease
IEL: intraepithelial lymphocyte
IFN-γ: interferon gamma
IL: interleukin
L: *Lactobacillus casei*
LP: lamina propria
LPL: lamina propria lymphocytes
PBL: peripheral blood lymphocytes
PBMC: peripheral blood mononuclear cell
TCR: T cell receptor
TGF: transforming growth factor
TNF-α: tumor necrosis factor-alpha
Treg: regulatory T cells
UC: ulcerative colitis
