# Supplementary figures and images for: CD4CD8αα Lymphocytes, A Novel Human Regulatory T Cell Subset Induced by Colonic Bacteria and Deficient in Patients with Inflammatory Bowel Disease
Source: PLoS Biol. 2014 Apr 8;12(4):e1001833. doi: 10.1371/journal.pbio.1001833 (PMC3979654; doi:10.1371/journal.pbio.1001833)

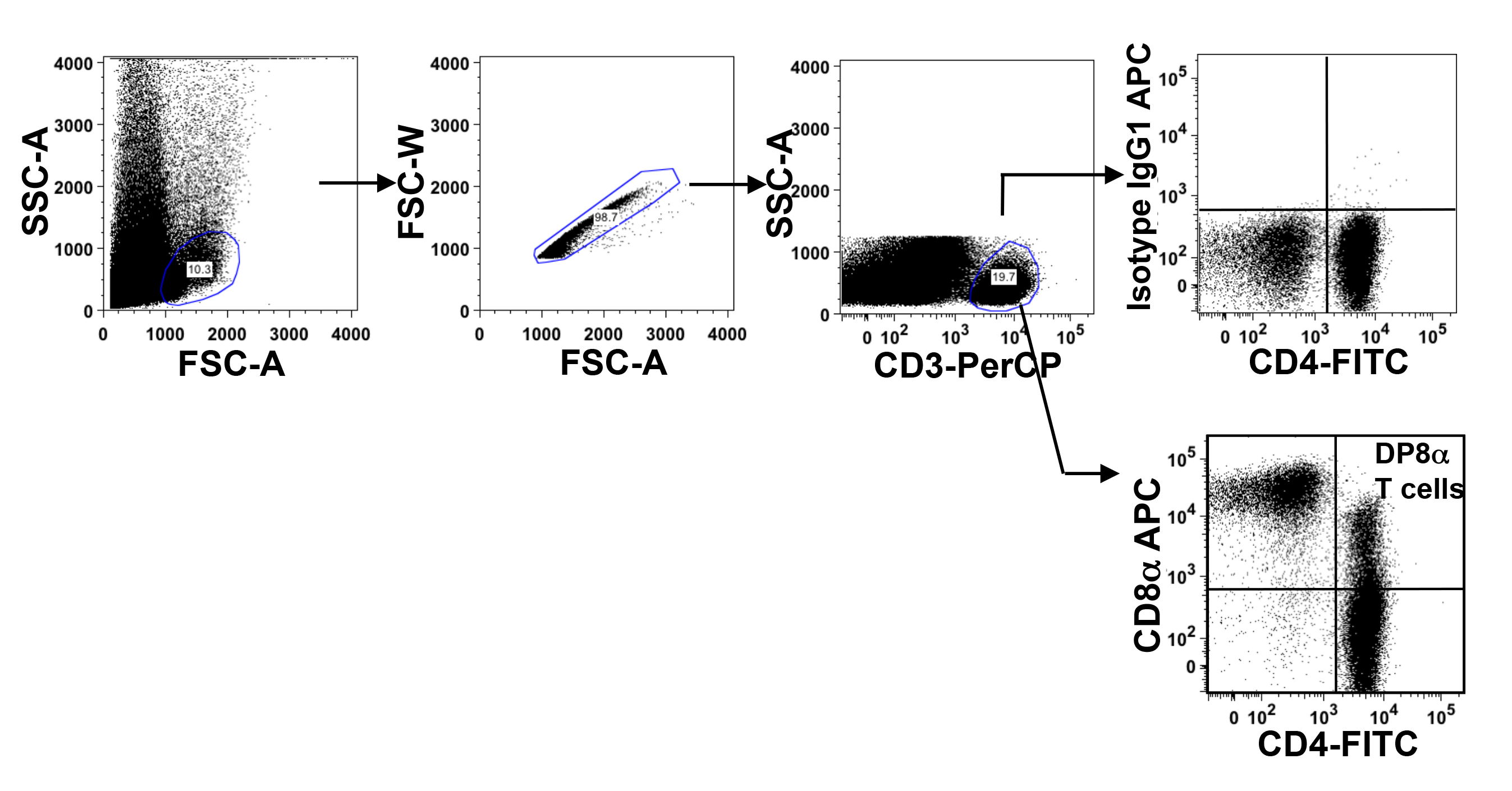

Supplement: Figure S1 — Marker combinations and gating options used for the quantification of DP8α cells among T lymphocytes obtained from freshly dissociated LP colonic samples. (TIF) [file pbio.1001833.s001.tif]

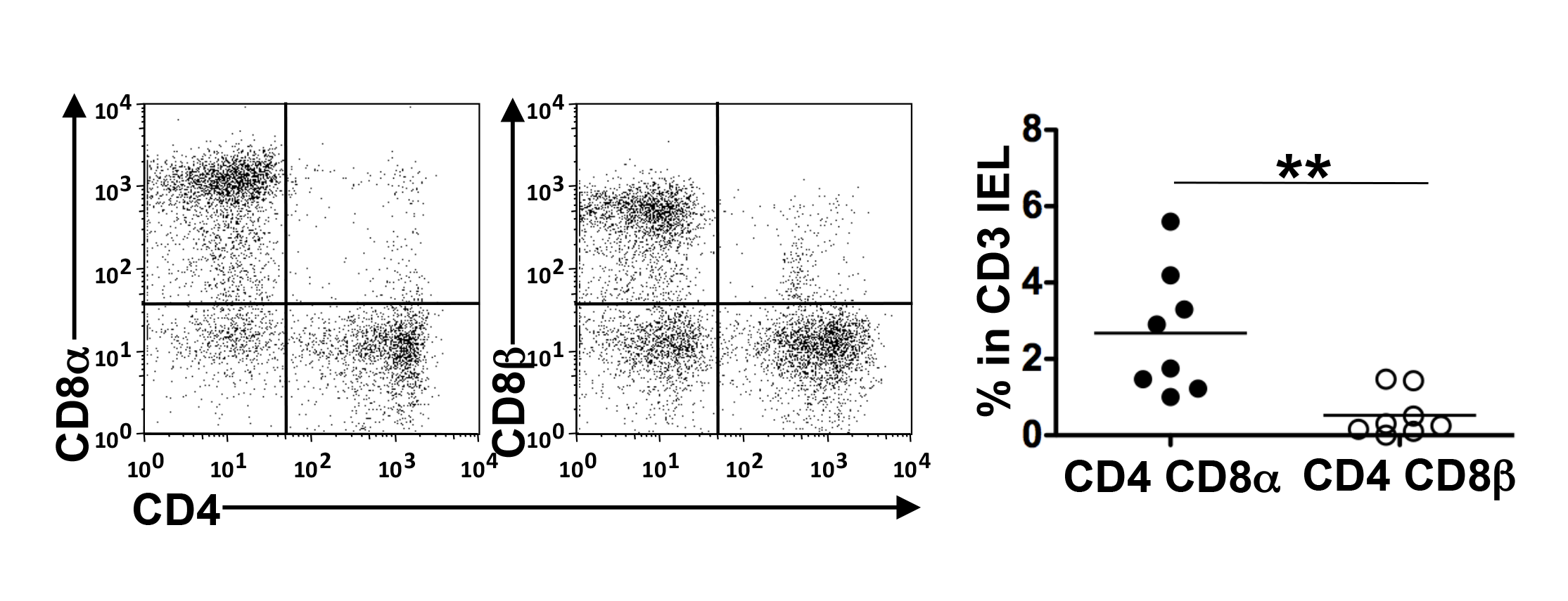

Supplement: Figure S2 — DP8α among the IEL of healthy colonic mucosa. Freshly dissociated IEL were analyzed by flow cytometry for the co-expression of CD4 and either CD8α or CD8β. Representative dot-plots and frequencies of CD4 T cells co-expressing the CD8α or CD8β among CD3 IEL from eight donors; **p<0.01 (paired t-test). (TIFF) [file pbio.1001833.s002.tiff]

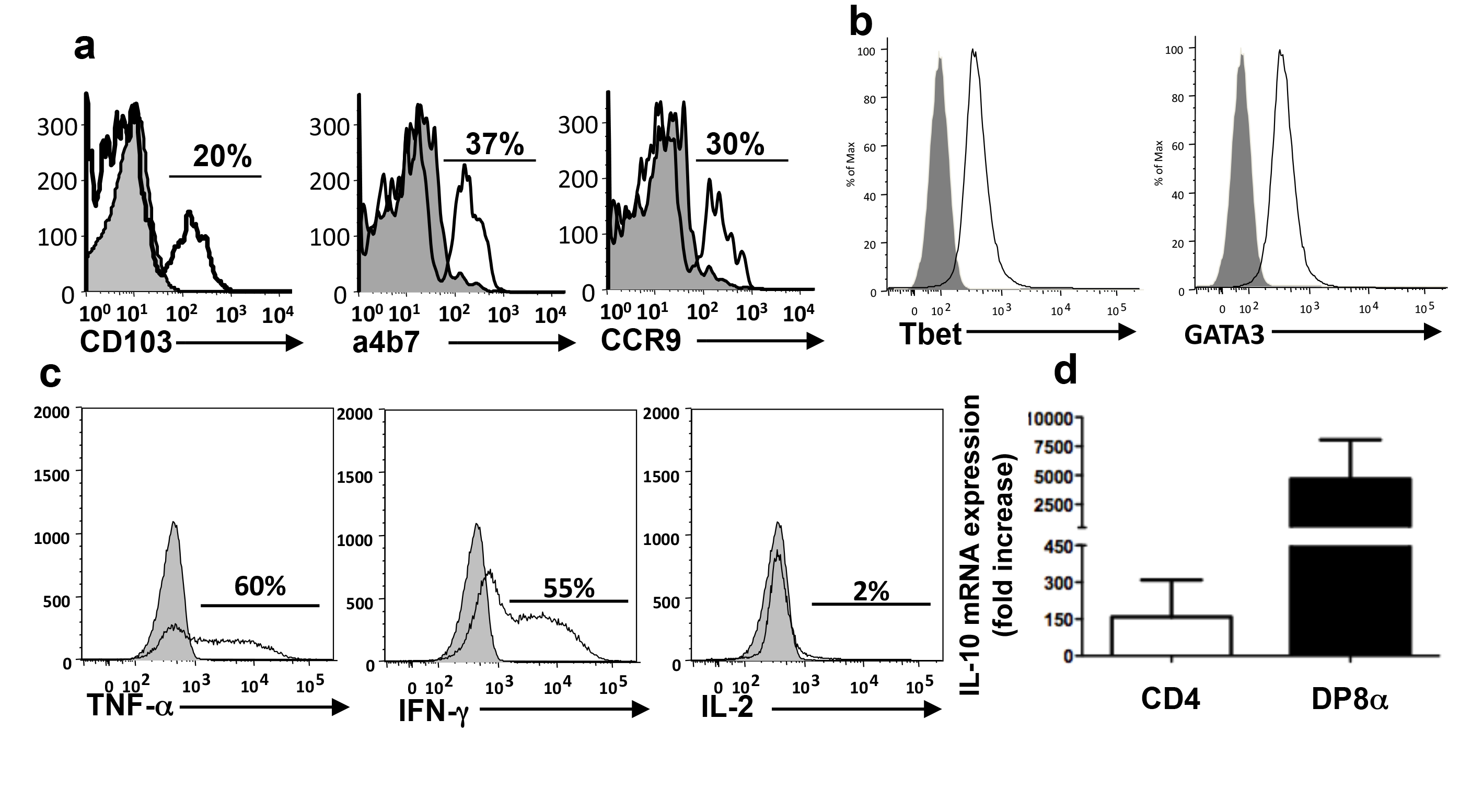

Supplement: Figure S3 — Flow cytometry analysis of the gut homing/localization markers and cytokine profile of DP8α LPL lymphocytes from healthy colonic mucosa. (a–c) Freshly dissociated CD3 LPL. (a) Expression of gut homing markers by DP8α LPL. (b) Expression of transcription factors, as in Figure 1e. (c) Cytokines secreted upon stimulation with anti-CD3 (as in Figure 1f). (d) IL-10 m RNA expression in DP8α and CD4 LPL lines (n = 3). (TIF) [file pbio.1001833.s003.tif]

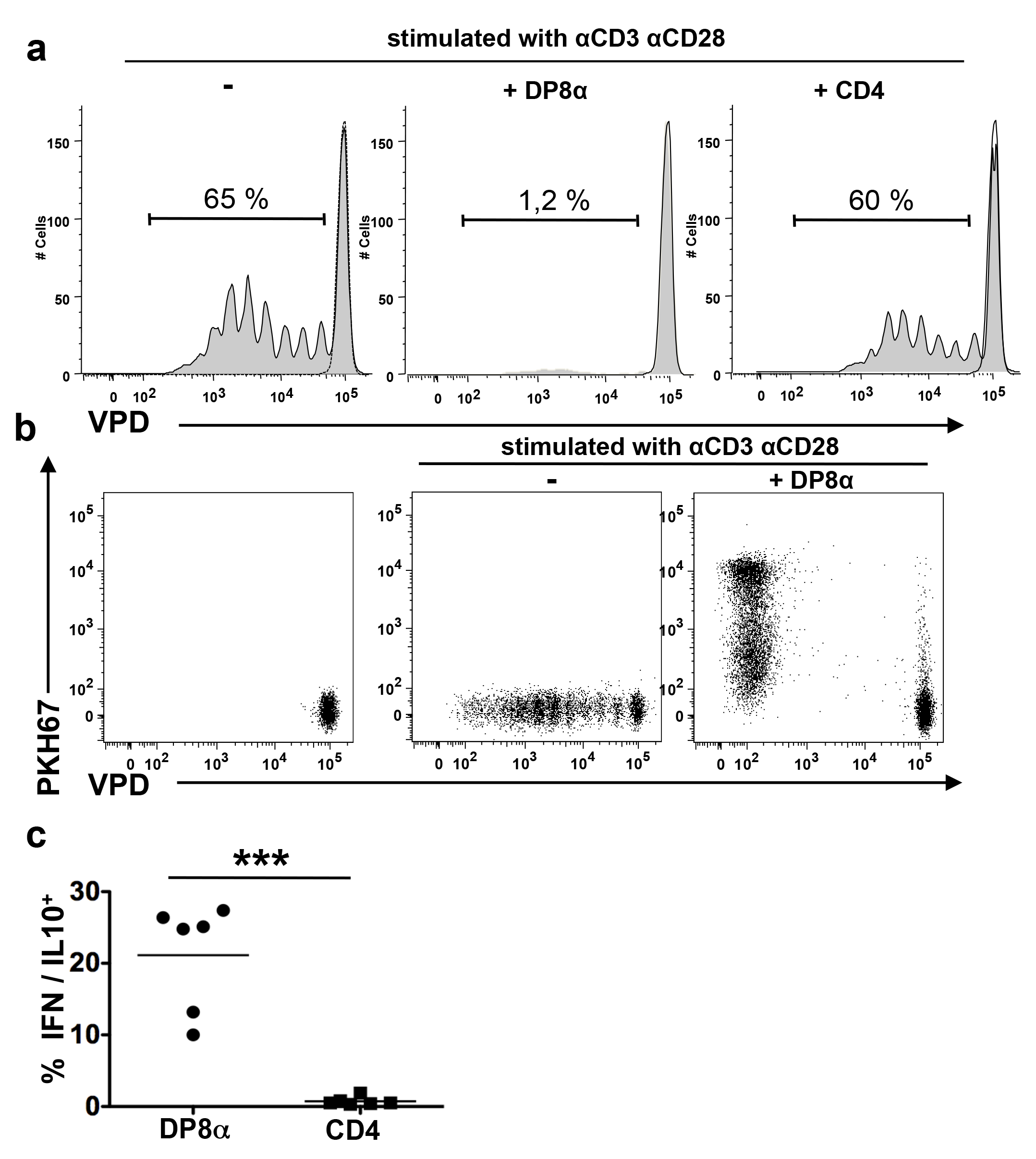

Supplement: Figure S4 — Lack of regulatory functions and F reactivity of CD4 LPL lines. (a) Flow cytometry figure showing the proliferation of CD4 lymphocytes is inhibited by DP8α LPL line C139 but not by its CD4 counterparts as measured by VPD dilution. (b) Dot plot showing the proliferative response of a DP8α LPL line (representative of four) upon stimulation with an anti-CD3 antibody (measured by PKH26 dilution) in a co-culture with VPD labelled CD4 T lymphocytes. (c) Flow cytometry analysis of the intracellular cytokine response of DP8α LPL lines (n = 3) and autologous CD4 LPL lines (n = 3), as in Figure 3b; ***p<0.001 (paired t-test). (TIF) [file pbio.1001833.s004.tif]

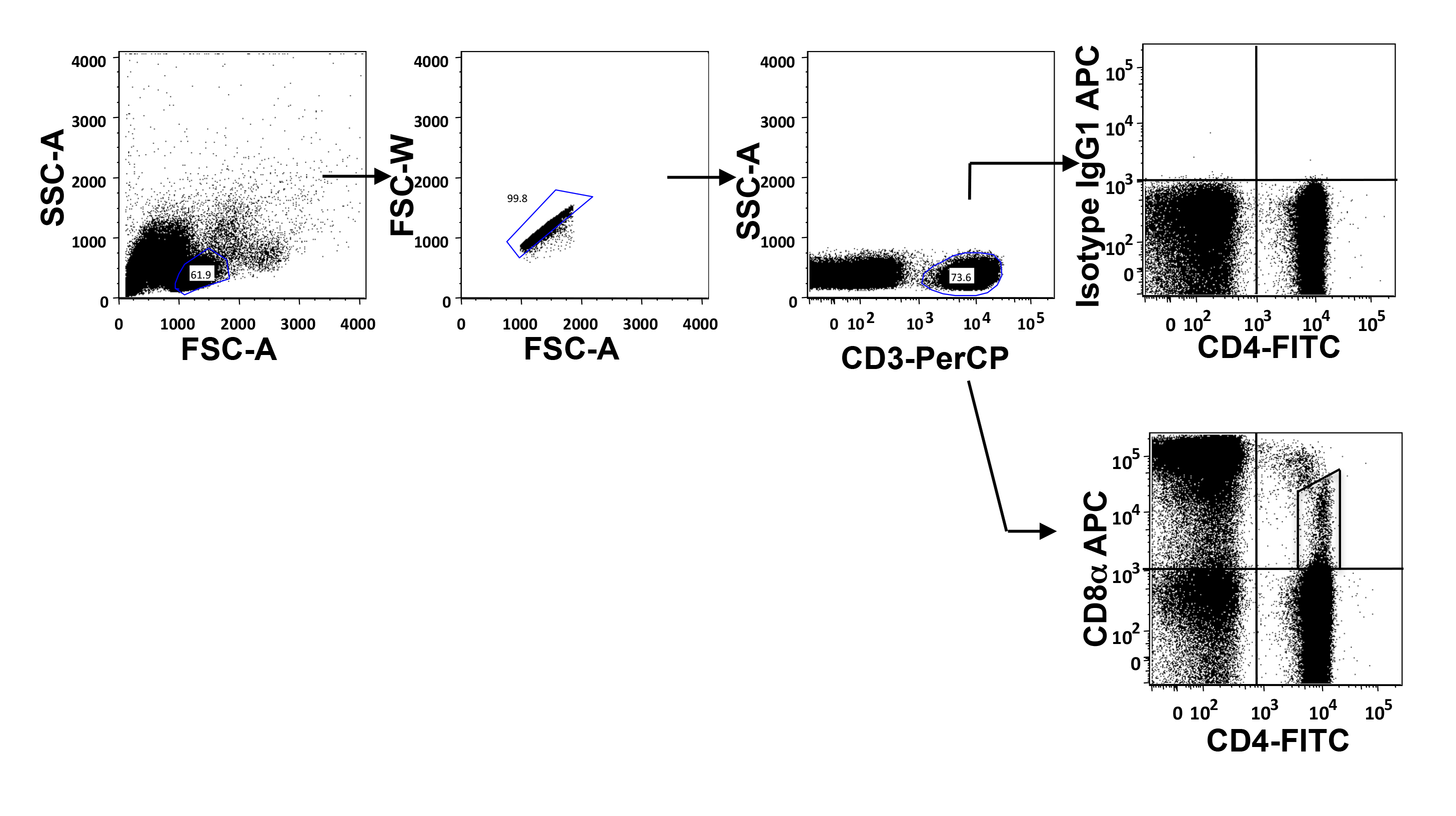

Supplement: Figure S5 — Marker combinations and gating options used for the quantification of DP8α cells among freshly-isolated PBMC. (TIF) [file pbio.1001833.s005.tif]
